# SESTD1 as a potential prognostic biomarker associated with tumor aggressiveness and immune infiltration in hepatocellular carcinoma

**DOI:** 10.1016/j.clinsp.2025.100796

**Published:** 2025-09-26

**Authors:** Ying Liu, Taoying Chen, Li Tang, Daqing Cheng, Ming Xu, Jiaoxiang Wu, Bingyan Liu, Hao Cheng, Bo Han, Yu Zhang, Sheng Cheng

**Affiliations:** a Hongqiao International Institute of Medicine, Tongren Hospital, Shanghai Jiao Tong University School of Medicine, Shanghai, China; b Clinical College of Traditional Chinese Medicine, Gansu University of Chinese Medicine, Lanzhou, China; c School of Food Science and Engineering, Guiyang University, Guizhou, China; d Rehabilitation Department, Tongren Hospital, Shanghai Jiaotong University School of Medicine, Shanghai, China; e Department of General Surgery, Tongren Hospital, Shanghai Jiao Tong University School of Medicine, Shanghai, China; f Department of Interventional Radiology, Tongren Hospital, Shanghai Jiao Tong University School of Medicine, Shanghai, China; g Rehabilitation Department, Shanghai Huayang Community Health Center, Shanghai, China; h Department of Otorhinolaryngology, Tongren Hospital, Shanghai Jiao Tong University School of Medicine, Shanghai, China

**Keywords:** SESTD1, Hepatocellular carcinoma, Diagnosis and prognosis, Proliferation and migration, Tumor immunity

## Abstract

**Background:**

Hepatocellular Carcinoma (HCC) is one of the most prevalent forms of malignancies worldwide, with a low overall survival rate and limited treatments. SESTD1 is upregulated in a variety of cancers and may serve as a potential prognostic indicator and therapeutic target. However, the expression pattern and functional relevance of SESTD1 in HCC are still unknown.

**Methods:**

SESTD1 expression and its prognostic value in HCC were evaluated by parsing multiple databases, including the Cancer Genome Atlas (TCGA), GEO databases, and Kaplan-Meier (KM) plotter. Gene Ontology (GO) and Gene Set Enrichment Analysis (GSEA) were conducted to investigate the potential biological functions and regulatory pathways of SESTD1. The relationship between SESTD1 expression and immune-infiltration levels was investigated in the Tumor Immune Estimation Resource (TIMER). RT-qPCR and Western blot analysis of tumor tissues were performed to verify SESTD1expression in HCC. CCK8, colony formation, and transwell assays were used to evaluate the influence of SESTD1 on HCC cell proliferation and migration.

**Results:**

The authors identified significantly elevated SESTD1 expression in Hepatocellular Carcinoma (HCC) tissues, which is associated with poor patient prognosis. Functional enrichment analysis revealed that SESTD1 regulates cell proliferation and the cell cycle, and in vitro experiments validated that silencing SESTD1 suppresses HCC cell proliferation and metastasis. Mechanistically, SESTD1 appears to modulate immune cell infiltration within the tumor microenvironment and shows a positive correlation with the expression of immune checkpoint molecules PD-1 and PD-L1.

**Conclusion:**

SESTD1 may serve as a diagnostic and prognostic marker, as well as a potential target for immunotherapy in HCC.

## Highlights

• SESTD1 is highly expressed in 15 cancer types and HCC patient tissues.
• Aberrant SESTD1 overexpression predicts worse prognosis in HCC patients.
• Silencing SESTD1 inhibits HCC cell proliferation, cell cycle and metastasis in vitro.
• SESTD1 positively related with immune infiltration and PD-1/PD-L1 expression in HCC.

## Introduction

Hepatocellular Carcinoma (HCC) is a frequently occurring deadly cancer and ranks as the fourth most common cause of cancer-related deaths globally.^1^ HCC patients only have a 10 %‒20 % 5-year survival rate.^2^ It is projected that over 10-million people would die from HCC by 2030, mostly due to late diagnosis and insufficient treatment options for the majority of patients.^3^ Although individuals with HCC have increasingly undergone extensive surgery, immunotherapy, and molecularly targeted therapies in recent years, their prognosis remains poor owing to the high risk of metastasis and recurrence associated with the disease.^4^^,^^5^ Therefore, the development of novel treatment targets for HCC and the identification of reliable biomarkers are urgently required.

SESTD1 (SEC14 and Spectrin Domains 1) is a protein that binds phospholipids and includes a SEC14-like domain for lipid binding, along with two Spectrin-repeat domains (SPEC1 and SPEC2).^6^ It was identified as a binding partner of TRPC4 and TRPC5, engaging with these transient receptor potential channels through their calmodulin- and inositol 1,4,5-trisphosphate receptor-binding domains^7^ SESTD1 exhibits a pronounced expression profile across a diverse array of human tissues, with particularly elevated levels observed in the brain, aortic tissue, adipose depots, and the testes.^6^ In a more recent study, Xia and colleagues used Whole-Genome Sequencing (WGS) to find that SESTD1 may be an oncogene in Non-Small Cell Lung Cancer (NSCLC), suggesting a close relationship between SESTD1 and tumor progression.^8^ However, studies on SESTD1 in tumors are still limited, especially in hepatocellular carcinoma, which has not been reported yet. Consequently, it is imperative to further explore the functions and mechanisms of SESTD1 in the progression of HCC.

In this present study, to systematically investigate the prognostic value of SESTD1 in HCC, the authors used the Cancer Genome Atlas (TCGA) and the Gene Expression Omnibus (GEO) databases to investigate the level of SESTD1 expression in HCC, validated its expression in clinical HCC tissues, and examined its correlation with the prognosis value of HCC patients using the Kaplan-Meier (KM) plotter. The authors further performed GO enrichment analysis and GSEA analysis and investigated the correlation between SESTD1 and immune-related cell infiltration to understand the biological mechanisms underlying the role of SESTD1 in the pathogenesis of HCC. Finally, cell proliferation and migration assays confirmed the functional role of SESTD1 in HCC.

## Materials and methods

### Data collection

The authors gathered RNA-seq transcriptomic data and corresponding clinical data from HCC patients (including 374 tumor tissues and 50 paracancerous tissues) in TCGA (https://portal.gdc.cancer.gov). The raw data were transformed into Transcripts Per Million (TPM) for further analyses. In addition, the GSE622232, GSE101685 and GSE121248 datasets from the GEO dataset were used to verify the differential expression of SESTD1. Evaluation of SESTD1 expression in hepatocellular carcinoma and its relationship with prognosis adhering to the Standards for Reporting of Diagnostic Accuracy (STARD) guidelines. Data analysis was conducted using R software.

### HCC tissue collection

After obtaining informed consent from all participating patients, 16 HCC tissues and the corresponding adjacent normal tissues were collected from 2020 to 2022 at Tongren Hospital of Shanghai Jiao Tong University School of Medicine. The samples were stored in liquid nitrogen for long-term preservation. This study was approved by the ethics board of Shanghai Tongren Hospital (nº 2020–035–01).

### Real-time quantitative PCR analysis

Total RNA of clinical samples and HCC cells was extracted with RNAisoPlus (9109, TaKaRa, Japan) based on the manufacturer’s protocol. The cDNA of samples was synthesized via HiScript II Q Select RT SuperMix for qPCR (R232–01, Vazyme, China). PCR amplifications were carried out using ChamQ Universal SYBR qPCR Master Mix (Q711–03, Vazyme, China). The primer sequences used for amplifying SESTD1 and β-actin in this study were as follows: primer for SESTD1 (forward: 5′-TTCCATTATGCCTCGAACAGAC-3′, reverse: 5′-ACGGTAAATCCTCTAGCCTTACA-3′) and primer for GAPDH (forward, 5′-GGAGCGAGATCCCTCCAAAAT-3′, reverse, 5′-GGCTGTTGTCATACTTCTCATGG-3′).

### Western blotting

Total proteins from HCC tissue samples and cells were extracted using the Radio-Immunoprecipitation Assay (RIPA) solution (P0013B, Beyotime, China), and protein concentrations were measured with the BCA protein assay kit (23,227, Thermo Fisher Scientific, USA). The proteins were then separated via SDS-PAGE and transferred onto PVDF membranes. Membranes were blocked with 5 % non-fat milk for 1 h at room temperature, then incubated with anti-SESTD1 (23,911–1-AP, 1:1000, Proteintech), anti-β-actin (T40104M, 1:2000, Abmart), and anti-GAPDH (M20006, 1:2000, Abmart) at 4 °C overnight. The membranes were incubated with a secondary antibody (1:2000) for 2 h at room temperature, and the bands were visualized using a chemiluminescent Horseradish Peroxidase (HRP) substrate (KF8001, Affinity, USA).

### Kaplan-Meier analysis

The correlation between SESTD1 expression and survival of patients with liver cancer was explored by Kaplan-Meier survival analysis, including Overall Survival (OS), Progression-Free Interval (PFI), and Disease-Specific Survival (DSS). Survival curves were performed in R (version 4.2.1) using the packages “survival” and “survmine”. A log-rank *p* < 0.05 was defined as statistically significant.

### Identification of differentially expressed genes

The authors implemented the R software package DEseq2(1.26.0)^9^ to identify Differentially Expressed Genes (DEGs) between the high and low SESTD1mRNA expression (cutoff value of 50 %) groups. An adjusted *p* < 0.05 and |log2Fold Change| > 1.5 were considered the threshold for identifying DEGs. Moreover, a heatmap was generated to show the top 10 downregulated and upregulated DEGs. Additionally, the Metascape online database^10^ (https://metascape.org/) was used to analyze the enrichment of SESTD1-related DEGs by pathway and process.

### Gene set enrichment analysis (GSEA)

The authors used the “clusterProfiler” R package to analyze pathway enrichment among SESTD1 groups with high and low expression levels.^11^ This analysis used c2.all.v7.2. symbols. Gmt curated from Molecular Signatures Database (MSigDB) as reference gene collection. The procedure was repeated 1000 times for each trait. An adjusted *p* < 0.05, absolute Normalized Enrichment Score (|NES|) > 1 and False Discovery Rate (FDR) < 0.25 were used as the criteria for statistical significance.

### Prognostic model construction and prediction

Receiver Operating Characteristic Curve (ROC) were generated using the R packages “pROC” and “ggplot” to estimate the diagnostic value of SESTD1. Nomograms with important clinical features and calibration plots were generated using the RMS R package and survival package. The survival package for multivariate Cox proportional hazard was used to evaluate prognostic factors.^12^ A bootstrap approach with 1000 resamples was used to calculate the Consistency index (C-index) as a measure of nomogram discrimination.

### Associations between SESTD1 expression and immune characteristics in HCC

The CIBERSORT algorithm was applied to present the relationships between SESTD1 and 22 types of tumor-infiltrating immune cells in HCC.^13^ Then, the single-sample Gene Set Enrichment Analysis (ssGSEA) method from the R package “GSVA” was conducted to estimate infiltration enrichment of 24 common immune cells.^14^ The authors also used the ggplot2 package to visualize the correlation between SESTD1 expression and immune cell infiltration, as well as the differences in immune cells between SESTD1 high and low expression groups.

### Cell culture

The human HCC cell lines MHCC-97H, SMMC7721, SK-Hep-1, HCCLM3, PLC-PRF-5 and HepG2 were purchased from Shanghai Institutes of Biological Sciences, Chinese Academy of Sciences (Shanghai, China). They were cultured in Dulbecco's Modified Eagle's Medium (DMEM) medium containing 10 % Fetal Bovine Serum (FBS), 100 U/mL streptomycin, and 100 mg/mL penicillin (all from Gibco/Invitrogen Technologies) at 37 °C with 5 % CO_2_ in a humidified incubator.

### Lentivirus transfection and stable cell line construction

To knock down SESTD1, the authors first cloned the shRNA sequence targeting SESTD1 (sh-SESTD1–1: CCGGTGGGATAAGAAAGTAACGCATCTCGAGATGCGTTACT TTCTTATCCCATTTTTG, sh-SESTD1–2: CCGGTTCCATCGGTTGATCCTGAAA CTCGAGTTTCAGGATCAACCGATGGAATTTTTG) into the pLKO.1-TRC lentiviral vector. Then, the lentiviral particles were produced by co-transfecting shRNA (sh-SESTD1) lentiviral vectors, the packaging plasmid psPAX2 (Addgene) and envelope plasmid pMD2.G (Addgene) into HEK 293T cells. The sh-SESTD1 lentivirus was transfected into the target cell line to knock down SESTD1, and non-specific shRNA lentivirus was used as a comparison. The transfected cells were selected by puromycin (2 μg/mL) for at least 1 week.

### Cell proliferation and migration assay

Cell proliferation assays were assessed using the Cell Counting Kit (CCK-8) (CK04, Dojindo, Japan). SESTD1 knocking-down stable cells in HCC cell lines (HepG2: 2 × 10^3^ cells per well& HCCLM3: 2 × 10^3^ cells per well) were seeded in 96-well plates with five duplicates. Add 10 µL of CCK-8 solution to each well and incubate the cells for 1 h at 37 °C in 5 % CO_2_. Absorbance was detected at 450 nm daily for 4 days. For colony formation assay, stable cells HepG2 and HCCLM3 (5000 cells per well) were seeded into 12-well plates with three repetitions and cultivated for 10-days. Cell colonies were stained using crystal violet dye, and the colonies were photographed and counted under a microscope. Migration assays were performed using a transwell chamber (3422, Corning, USA) according to the manufacturer’s protocol. A total of 3 × 10^4^ HCC cells in serum-free medium were placed in the upper chamber, and the lower chamber received medium containing 10 % FBS. Following incubation for 48 h at 5 % CO_2_ and 37 °C, cells on the underside were fixed with 4 % paraformaldehyde and stained using 0.5 % crystal violet. The penetrated cells were counted under a light microscope.

### Statistical analysis

The analyses of experimental data were carried out using GraphPad Prism 8.0.1 software. With a two-sided Student’s unpaired *t*-test, p-value < 0.05 was considered significant.

## Results

### SESTD1 is expressed in cancer tissues and localized in tumor cell lines

**Fig. 1:**
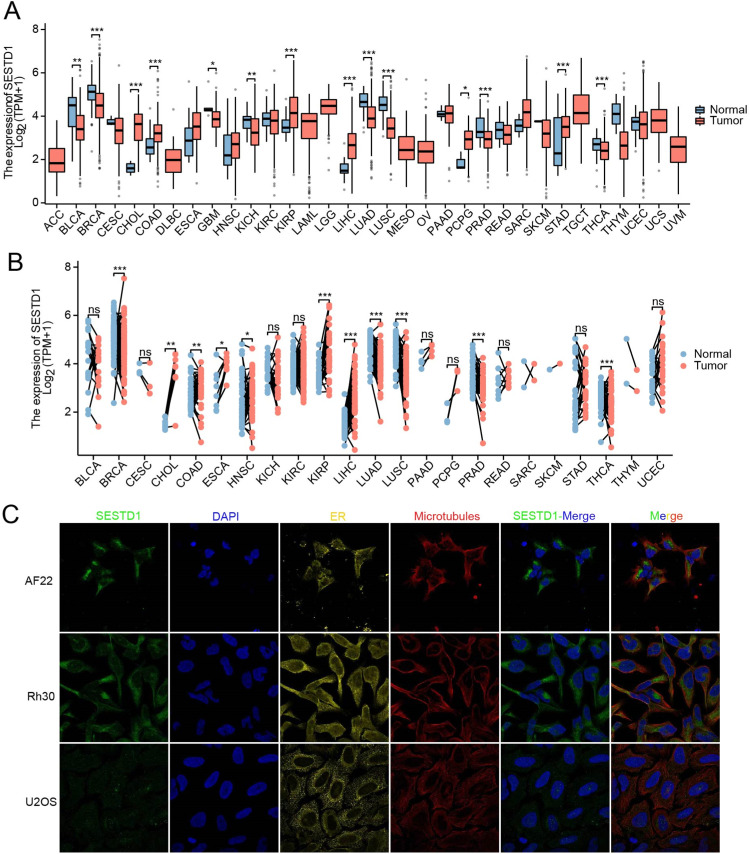
SESTD1 expressed in pan-cancer and localization in tumor cell lines. (A) The expression of SESTD1 in different tumor types; (B) SESTD1 expression in cancer vs. paired adjacent tissues. (C) Subcellular distribution of SESTD1 from the HPA database (* *p* < 0.05, ** *p* < 0.01, and *** *p* < 0.001).

To explore the possible roles of SESTD1 in carcinogenesis, the authors investigated the differential expression of SESTD1 in multiple cancer types using TCGA data. The present analysis showed that a high expression of SESTD1 was observed in all six tumors: Cholangiocarcinoma (CHOL), Colon Adenocarcinoma (COAD), Kidney Renal Papillary Cell Carcinoma (KIRP), Liver Hepatocellular Carcinoma (LIHC), Pheochromocytoma and Paraganglioma (PCPG), and Stomach Adenocarcinoma (STAD). In comparison, a low SESTD1 expression was noticed in seven tumors: Bladder Urothelial Carcinoma (BLCA), Breast Invasive Carcinoma (BRCA), Kidney Chromophobe (KICH), Lung Adenocarcinoma (LUAD), Lung Squamous Cell Carcinoma (LUSC), Prostate Adenocarcinoma (PRAD), and Thyroid carcinoma (THCA) (Fig. 1A). In paired adjacent tissues, the authors observed the high level of SESTD1 expression in five tumors: CHOL, Esophageal Carcinoma (ESCA), KIRP, LIHC, and THCA, and a low SESTD1 expression in six tumors: BRCA, COAD, Head and Neck Squamous Cell Carcinoma (HNSC), LUAD, LUSC, and PRAD (Fig. 1B). To characterize the specific intracellular localization of SESTD1 protein, the authors obtained the immunofluorescence of SESTD1 protein images from the Human Protein Atlas (HPA). It showed that SESTD1 protein was mainly localized in the cytosol, and colocalization with endoplasmic reticulum or microtubules in three tumor cell lines including human neuroepithelial stem cell line AF22, human rhabdomyosarcoma cell line Rh30, and human osteosarcoma cell line U2OS (Fig. 1C). Overall, the pan-cancer analysis revealed a significant upregulation of SESTD1 mRNA in liver cancer samples, leading to the hypothesis that SESTD1 may be involved in the malignant progression of Hepatocellular Carcinoma (HCC). In follow-up research, the authors focused on the role of SESTD1 in HCC.

### Abnormally high expression of SESTD1 in HCC

**Fig. 2:**
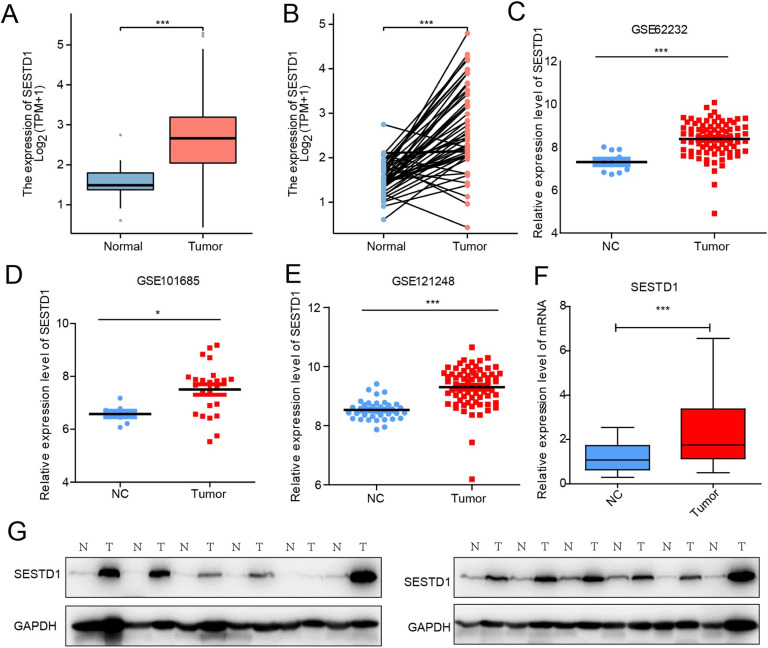
SESTD1 expression in HCC patients. (A) SESTD1 expression levels in 374 HCC samples and 50 normal samples; (B) SESTD1 expression levels in HCC and paired normal tissues (*n* = 50); (C‒E) Expression of SESTD1 was detected in three different GEO databases; (F) SESTD1 mRNA expression in HCC tissues and paired normal liver tissues (*n* = 16); (G) Western blotting detect SESTD1 protein level in HCC and paired normal tissues (*p < 0.05, ** *p* < 0.01, and *** *p* < 0.001).

Further comparison of SESTD1 expression in 374 HCC samples and 50 paracancer samples in the TCGA dataset revealed that SESTD1 expression was significantly higher in the HCC samples (*p* < 0.001) (Fig. 2A). Specifically, in paired specimens, SESTD1 expression in 50 HCC samples was significantly higher than in matched paracancerous samples (*p* < 0.001) (Fig. 2B). Furthermore, the authors evaluated SESTD1 expression in three GEO datasets (GSE62232, GSE101685, and GSE121248) and confirmed its high expression level in HCC. (Fig. 2C‒E). To verify the aberrant expression of SESTD1 in HCC, the authors assessed the mRNA and protein expression levels of SESTD1 in 16 HCC samples and their corresponding normal tissues. The RT-qPCR and western blot staining analysis revealed the abnormally high expression of SESTD1 in HCC (Fig. 2F and G). These validated data illustrate that SESTD1 may be a promising biomarker in the progression of HCC.

### Correlation analysis between SESTD1 expression and clinical characteristics

**Fig. 3:**
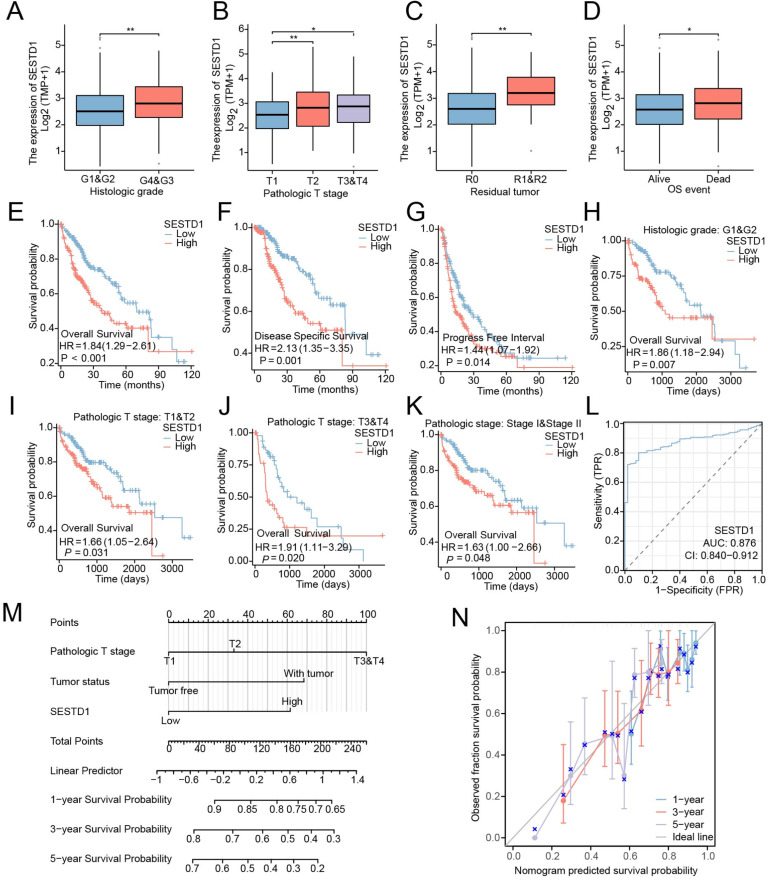
Clinicopathological characteristics and prognostic value of SESTD1 in HCC. (A‒D) Associations between SESTD1 expression and clinicopathological parameters, including Histological grade, Pathologic T-stage, Residual tumor, OS event; (E‒G) Kaplan‐Meier survival analysis for OS, DSS, and PFI of HCC patients with high and low expression levels of SESTD1; (H‒K) Subgroup analyses of OS; (L) Receiver Operating Characteristic analysis (ROC) of SESTD1 in HCC; (M) A nomogram that integrates SESTD1 and other prognostic factors in HCC; (N) Calibration plots validating the effectiveness of nomograms for OS (* *p* < 0.05, ** *p* < 0.01, and *** *p* < 0.001).

Since the expression level of SESTD1 is intimately related to HCC development, the authors examined SESTD1 expression in distinct patient groups based on clinical characteristics, including histological grade, T-stage, residual tumor, and Overall Survival (OS) event in the TCGA database. The present data confirmed high SESTD1 expression was correlated with higher histological grade (G3 & G4 vs. G1 & G2, *p* < 0.01) (Fig. 3A), higher stage-T (T2 vs. T1, *p* < 0.01 and T3 & T4 vs. T1, *p* < 0.05) (Fig. 3B), and more residual tumor (R2 & R2 vs. R0, *p* < 0.01) (Fig. 3C). The analysis also revealed that SESTD1 expression was significantly correlated with OS event (Fig. 3D). These results indicated that a high SESTD1 expression might positively correlate with HCC progression.

### Prognostic potential of SESTD1 in HCC

According to research on SESTD1 expression in HCC and its correlation with clinical characteristics, high SESTD1 expression may be a useful marker for determining the cancerousness of HCC. All patients were divided into a high expression group and a low expression group according to the median expression level of SESTD1. The authors explored the correlation of SESTD1expression levels with the prognosis of HCC patients using the Kaplan-Meier survival analysis. The results showed that high expression of SESTD1 was highly correlated with poor OS, Disease Specific Survival (DSS), and Progression Free Interval (PFI) (Fig. 3E and F). The authors also performed subgroup analyses of OS, including histological grade (G1 & G2), pathologic T stage (T1 & T2 and T3 & T4), and pathologic stage (stage I & stage II) in relation to SESTD1 expression. The findings indicated that the OS of the subgroup of HCC patients with elevated SESTD1 was markedly worse (Fig. 3H‒K). By constructing a Receiver Operating Characteristic (ROC) curve, the authors found that SESTD1 had a significant diagnostic value for HCC with an Area Under the Curve (AUC) of 0.876 (95 % CI 0.840‒0.912) (Fig. 3L). In addition, the authors created a nomogram of OS to integrate SESTD1 expression and other independent clinical risk factors, including pathologic T-stage and tumor status (Fig. 3M). A higher point on the nomogram represented a worse prognosis, and the calibration curve was employed to assess the nomogram’s performance of SESTD1. The C-index for OS was 0.646 with 1000 bootstrap replicates, indicating that the nomogram model for OS demonstrated good consistency in HCC patients (Fig. 3N).

### Functional enrichment analysis of DEGs in HCC

**Fig. 4:**
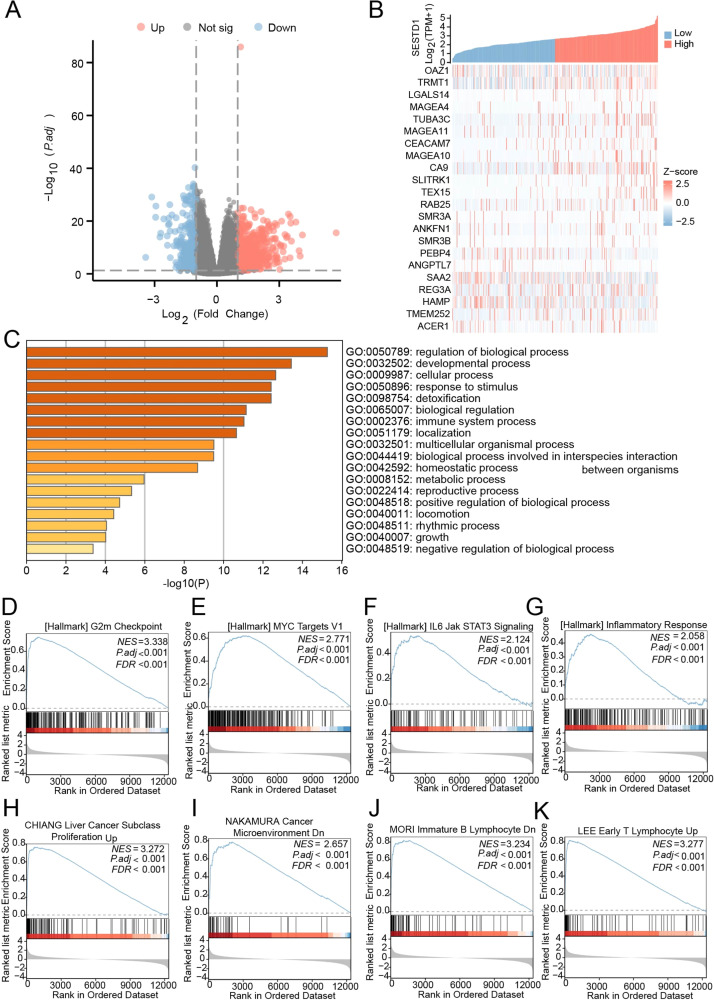
Functional enrichment analysis of DEGs in HCC. (A) Volcano Plot of DEGs showing 785 genes up (red) and 303 down-regulated genes (blue); (B) Heatmap showing the top-10 upregulated and downregulated DEGs; (C) Top-20 terms of GO enrichment analysis. (D‒K) GSEA results showing differential enrichment of genes in HCC cases with high SESTD1 expression, including G2m Checkpoint, MYC Targets, IL6 Jak STAT3 Signaling, Inflammatory Response, Liver Cancer Subclass Proliferation Up, Cancer Microenvironment, Immature B Lymphocyte, and Early T Lymphocyte Up.

The authors examined the gene expression patterns in the HCC tissues of patients with high and low SESTD1 expression to gain a better understanding of how this protein controls the course of the disease. A total of 1088 Differentially Expressed Genes (DEGs) were identified, including 785 that were downregulated and 303 that were upregulated (|Log2-fold change| > 1.0, adjusted *p* < 0.05) (Fig. 4A). The heatmap was generated to show the top 10 upregulated and downregulated DEGs (Fig. 4B). These DEGs were enriched in items such as cellular process, reproductive process, regulation of biological process, immune system process and growth in the GO enrichment study (Fig. 4C). GSEA enrichment analysis shown that upregulated SESTD1 expression in HCC was associated with G2m Checkpoint, MYC Targets, IL6 Jak STAT3 Signaling, Inflammatory Response, Liver Cancer Subclass Proliferation Up, Cancer Microenvironment, Immature B Lymphocyte, and Early T Lymphocyte Up (Fig. 4D‒K).

### The relationship between SESTD1 expression and infiltrating immune cells

**Fig. 5:**
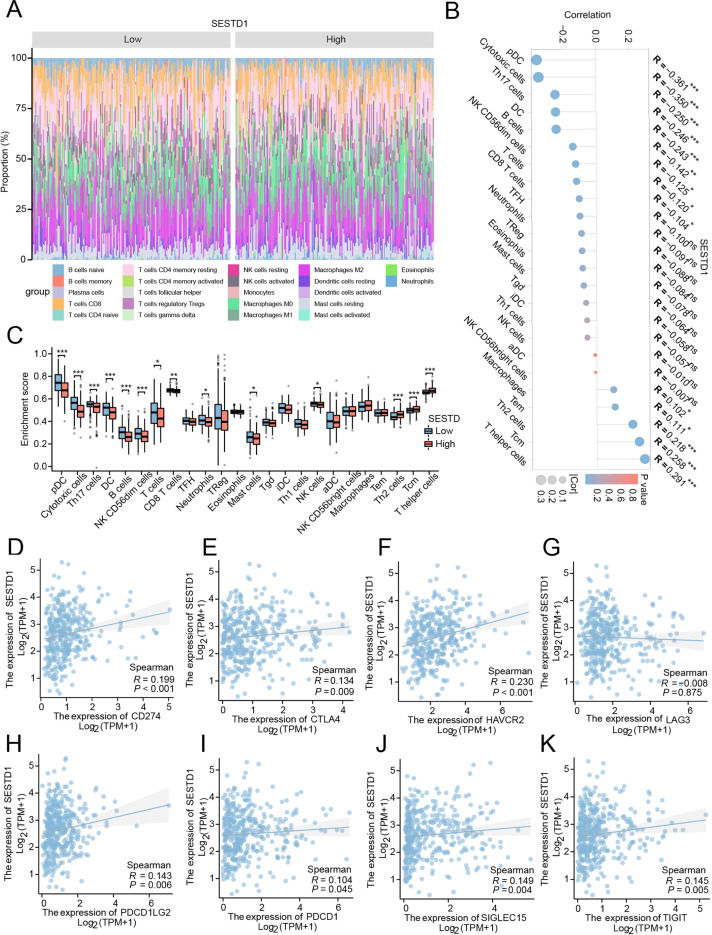
Correlation of SESTD1 expression with immune infiltration in HCC. (A) The distribution of twenty-two tumor-infiltrating immune in HCC samples using the CIBERSORT algorithm; (B‒C) Relationship between SESTD1 expression and infiltration of 24 immune cell types; (D‒K) The correction between SESTD1 expression and eight ICGs (*p < 0.05, **p < 0.01, and ***p < 0.001).

Since high SESTD1 expression has been linked to tumor immunity and worsening prognoses in HCC, the authors explored the “Estimate” algorithm to evaluate the relationships between SESTD1 and tumor-infiltrating immune cells in HCC (Fig. 5A). This analysis found that SESTD1 expression markedly positively correlated with infiltrating levels of T helper cells, Tcm, Th2 cells, Tem, and Macrophage in HCC (Fig. 5B). In contrast, SESTD1 expression was strongly negatively correlated with the level of pDC, Cytotoxic cells, Th17 cells, DC, B cells, NK CD56dim cells, T-cells, CD8 T-cells, and THF infiltration in HCC (Fig. 5C). Additionally, the authors used the Spearman test to examine the relationship between SESTD1 expression and eight Immunological Checkpoint Genes (ICGs). The authors found that the expression of SESTD1 positively correlated with CD274 (*r* = 0.199, *p* < 0.001), CTLA4 (*r* = 0.134, *p* = 0.009), HAVCR2 (*r* = 0.230, *p* < 0.001), PDCD1LG2 (*r* = 0.143, *p* = .006), PDCD1 (*r* = 0.104, *p* = 0.045), SIGLEC15 (*r* = 0.149, *p* = 0.004), and TIGIT (*r* = 0.145, *p* < 0.005) (Fig. 5D‒K).

### Knocking down SESTD1 expression inhibited the proliferation and migration of HCC cells

**Fig. 6:**
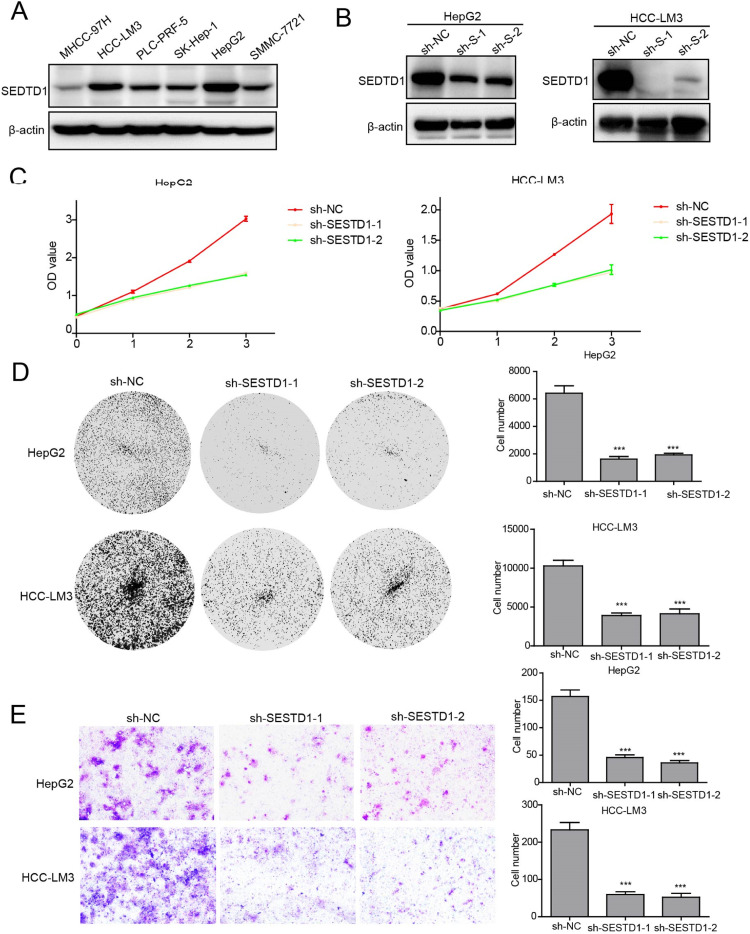
Deletion of SESTD1 inhibits the proliferation and migration of HCC cell lines. (A) Western blot detection of SESTD1 expression in six HCC cell lines; (B) Western blot validation of SESTD1 knockdown in HCCLM3 and HepG2 cell lines; (C) CCK8 analysis showing SESTD1 knockout inhibited the proliferation of HCCLM3 and HepG2 cell lines; (D‒E) Colony formation and transwell assays showed that silencing SESTD1 greatly reduced proliferation and migration ability of HCCLM3 and HepG2 cell lines (*p < 0.05, **p < 0.01, and ***p < 0.001).

The present bioinformatics data analysis indicates that excessively high SESTD1 expression is strongly associated with a poor prognosis for HCC and the infiltration of many immune cells, which raises the possibility that SESTD1 plays a role in accelerating the malignant development of HCC. To verify the aggressive role of SESTD1 in HCC, the authors manipulated SESTD1 expression by shRNA in two SESTD1-overexpressing HCC cell lines, HCCLM3 and HepG2, to assess the regulatory role of SESTD1 on HCC cell function (Fig. 6A and B). CCK8 assay, colony formation, and transwell experiment suggested that inhibition of SESTD1 expression significantly reduced proliferative and metastatic potential of HCCLM3 and HepG2 (Fig. 6C‒E). These results demonstrate that SESTD1 may function as an oncogene in HCC.

## Discussion

The most prevalent kind of hepatocellular carcinoma has a dismal prognosis because there are currently few effective therapy options.^15^ Finding novel approaches to enhance clinical care and diagnostic precision is, therefore, essential. In the current study, the authors parsed the TCGA and GEO databases and found that SESTD1 expression was increased abnormally in HCC, and further confirmed this high expression pattern of SESTD1 in the clinical HCC samples. Abnormally high SESTD1 expression is strongly associated with pathological stage, T-stage, histological grade, OS events, tumor status, AFP, and poor prognosis of HCC. More importantly, the AUC score of the ROC curve analysis revealed a reliable diagnostic value of SESTD1 expression for HCC. These findings lead us to draw the conclusion that SESTD1 expression could serve as a potential diagnostic biomarker in HCC.

To further elucidate the underlying potential functions and mechanisms of SESTD1 associated with HCC, the authors identified 1088 DEGs by comparing the two groups based on the high and low SESTD1 expression and performed GO enrichment analysis. The results suggested that SESTD1 was mainly related to the regulation of biological processes, cellular processes, growth, and immune system processes. In support of this, the authors conducted SESTD1 knockdown in two HCC cell lines and confirmed that SESTD1 knockdown inhibits HCC cell proliferation and migration. The bioinformatics analysis and validation experiments suggest a functional role for SESTD1 in promoting the proliferation and migration of HCC cells, which provides a new inspiration for the treatment of HCC targeting SESTD1.

Further GSEA analysis revealed that the SESTD1-high phenotype was linked to the G2/M checkpoint, MYC target gene sets, and liver cancer proliferation. The G2/M checkpoint plays a critical role in the early stages of HCC, and its dysregulation may enable cancer cells to evade DNA repair, thereby accelerating tumor proliferation.^16^ Recent research has shown that MYC, a critical oncogene in hepatocellular carcinoma, can drive tumor initiation and progression by modulating cell cycle, metastasis, and angiogenesis.^17^^,^^18^ Therefore, SESTD1 expression may be essential for HCC tumorigenesis. Interestingly, GO enrichment analysis indicated SESTD1 was correlated with immune system processes, and GSEA also indicated that SESTD1 might play a role in tumor microenvironment and immune regulation in HCC, including Cancer Microenvironment, IL6 Jak STAT3 Signaling, Inflammatory Response, Immature B Lymphocyte, and Early T Lymphocyte Up. The authors further found that high SESTD1 expression is associated with high abundance of immune infiltration, including T helper cells, Th2 cells, and Macrophages in HCC. T helper cells, particularly Th2 cells, may inhibit antitumor immune responses by releasing anti-inflammatory cytokines like IL-4 and IL-10, which can promote the growth and spread of tumor cells.^19^^,^^20^ Macrophages, as one of the most abundant types of tumor-infiltrating immune cells, have been extensively examined and validated for their cellular typing and vital involvement in tumorigenesis, progression, and metastasis.^21^, ^22^, ^23^, ^24^ Recent reports have confirmed the infiltration of macrophages to act synergistically with T-cells to encourage immunosuppression and the development of HCC.^25^ Based on these results and those of prior studies, the authors hypothesize that SESTD1 may be involved in the oncogenesis and progression of HCC by regulating immune-related pathways that enhance immune infiltration.

In recent years, immune checkpoint inhibition has evolved into the primary tumor immunotherapy technique.^26^^,^^27^ Checkpoint blockade therapy targeting PD-1/PDL-1 axis is commonly employed for treating various malignant tumors, including HCC.^28^^,^^29^ Targeting the PD-1/PD-L1 axis has recently shown unheard-of effectiveness in treating HCC, but poor remission rates pose serious problems that need to be resolved. Not all HCC patients have the same limiting factors for antitumor immunity, and blocking the PD-1/PD-L1 axis by itself is not enough to generate a potent antitumor immune response.^30^^,^^31^ Therefore, exploring new immunotherapeutic targets or combination therapy with conventional chemotherapy and PD-1 may benefit more patients with HCC. Interestingly, the authors noted a significant association between high SESTD1 expression and the levels of PD-1, PD-L1, and CTLA-4 in HCC. SESTD1 may prove to be a potential immunotherapeutic target for HCC with additional immunotherapeutic validation in the future.

Although this work has advanced understanding of the regulatory mechanism of SESTD1 in HCC, several key predictions require further functional validation., There were limitations regarding the size and quality of the clinical samples. The prognostic significance of SESTD1 expression in HCC should be further validated in larger-scale clinical samples. Future work is needed to conduct in vivo and in vitro studies to explore the underlying mechanisms by which SESTD1 regulates the immune infiltration of HCC.

In conclusion, these findings suggested that SESTD1 is overexpressed in HCC with hypomethylation levels. Both upregulation of SESTD1 expression and reduction in its methylation levels are correlated with poor prognosis in HCC. Functional experiments showed that knockdown of SESTD1 inhibited the proliferation and migration of HCC cells. Additionally, mechanistic exploration analysis revealed that SESTD1 mediated immune cell infiltration in HCC. This study demonstrates that SESTD1 could serve as a diagnostic and prognostic marker for HCC and implies that it might make a promising immunotherapy target.

## Ethics approval

This study was approved by Tongren Hospital of Shanghai Jiao Tong University School of Medicine (nº 2020–035–01). Before sample collection, written informed consent was provided by all participating patients.

## Data availability

All data generated or analyzed during this study are included in this published article; further inquiries can be directed to the corresponding author/s.

## Authors’ contributions

Y.L. and T.C.: Methodology, Validation, Data Curation, Software; L.T.: Methodology, Validation, Funding Acquisition; D.C., B.L., and M.X.: Data Curation, Visualization; J.W. and H.C.: Investigation, Writing-Review & Editing; B.H.: Conceptualization, Writing-Original Draft, Validation; Y.Z.: Conceptualization, Writing-Original Draft, Supervision; S.C.: Conceptualization, Writing-Original Draft, Supervision, Funding Acquisition.

## Funding

This work was supported by the National Natural Science Foundation of China (82002495), Research Fund of Shanghai Tongren Hospital (TRKYRC-xx202205, TRKYRC-xx202212, TRKYRC-xx202213), Fundamental Research Funds for the Central Universities (YG2025QNB53, YG2022QN117), and Research Fund of Key Laboratory for Translational Research and Innovative Therapeutics of Gastrointestinal Oncology (ZDSYS-2021-04).

## Conflicts of interest

The authors declare no conflicts of interest.

## Appendix Supplementary materials

Image, application 1
