# Supplementary material for: SESTD1 as a potential prognostic biomarker associated with tumor aggressiveness and immune infiltration in hepatocellular carcinoma
Source: Clinics (Sao Paulo). 2025 Sep 26;80:100796. doi: 10.1016/j.clinsp.2025.100796 (PMC12510035; doi:10.1016/j.clinsp.2025.100796)
Supplement: Supplementary file 1 [file mmc1.docx]

**CLINICS-D-25-00930_ Supplementary Material**

**Supplementary Figure 1** The original blot of Western blotting. Prior to hybridization with antibodies during the blotting procedure, the non-target band portions of the original blot are removed to conserve the antibodies.
